# Characterization of Endocannabinoid-Metabolizing Enzymes in Human Peripheral Blood Mononuclear Cells under Inflammatory Conditions

**DOI:** 10.3390/molecules23123167

**Published:** 2018-12-01

**Authors:** Brittany N. Szafran, Jung Hwa Lee, Abdolsamad Borazjani, Peter Morrison, Grace Zimmerman, Kelly L. Andrzejewski, Matthew K. Ross, Barbara L.F. Kaplan

**Affiliations:** 1 Center for Environmental Health Sciences, Department of Basic Sciences, College of Veterinary Medicine, Mississippi State University, Starkville, MS 39759, USA; bns267@msstate.edu (B.N.S.); junghwa0519@gmail.com (J.H.L.); aborazjani@cvm.msstate.edu (A.B.); 2 Department of Neurology, University of Rochester, Rochester, NY 14627, USA; Peter_Morrison@URMC.Rochester.edu (P.M.); Grace_Zimmerman@URMC.Rochester.edu (G.Z.); klandrze@buffalo.edu (K.L.A.); 3 Jacobs School of Medicine and Biomedical Sciences, University of Buffalo, Buffalo, NY 14211, USA

**Keywords:** endocannabinoids, MAGL, CES, Huntington’s disease

## Abstract

Endocannabinoid-metabolizing enzymes are downregulated in response to lipopolysaccharide (LPS)-induced inflammation in mice, which may serve as a negative feedback mechanism to increase endocannabinoid levels and reduce inflammation. Increased plasma levels of the pro-inflammatory cytokine interleukin-6 (IL-6) and decreased fatty acid amide hydrolase (FAAH) activity in peripheral lymphocytes from individuals diagnosed with Huntington’s disease (HD) suggests that a similar negative feedback system between inflammation and the endocannabinoid system operates in humans. We investigated whether CpG- (unmethylated bacterial DNA) and LPS-induced IL-6 levels in peripheral blood mononuclear cells (PBMCs) from non-HD and HD individuals modulated the activities of endocannabinoid hydrolases monoacylglycerol lipase (MAGL) and carboxylesterase (CES). Baseline plasma IL-6 levels and 2-arachidonoylglycerol (2-AG) hydrolytic activity in PBMC lysates were not different in HD and non-HD individuals. Inhibition of MAGL and CES1 activity in PBMCs using the inhibitors JZL184 and WWL113, respectively, demonstrated that MAGL was the dominant 2-AG hydrolytic enzyme in PBMCs, regardless of disease state. Correlative analyses of 2-AG hydrolytic activity versus enzyme abundance confirmed this conclusion. Flow cytometric analysis of PBMCs showed that MAGL and CES1 were primarily expressed in monocytes and to a lesser extent in lymphocytes. In conclusion, these data suggest that IL-6 did not influence 2-AG hydrolytic activity in human PBMCs; however, monocytic MAGL was shown to be the predominant 2-AG hydrolytic enzyme.

## 1. Introduction

The endocannabinoid system comprises arachidonoyl-containing endocannabinoids, their cognate G-protein coupled receptors, and biosynthetic and catabolic enzymes that regulate the levels of these lipid signaling molecules. Engagement of the cannabinoid 1 or 2 receptors by plant-derived cannabinoids or endogenous cannabinoids is known to produce anti-inflammatory effects [1,2]. 2-Arachidonoylglycerol (2-AG) and anandamide (AEA) are the best characterized endocannabinoids and are hydrolytically catabolized by monoacylglycerol lipase (MAGL) and fatty acid amide hydrolase (FAAH), respectively, yielding pro-inflammatory arachidonic acid (AA) [3,4]. Other enzymes known to hydrolyze and inactivate 2-AG include carboxylesterases (CES1 and CES2 in human, Ces2g in mice; human and mouse gene symbols for carboxylesterases are CES and Ces, respectively), α,β-hydrolase domain (ABHD) 6, and ABHD12 [5,6,7]. It should be noted that FAAH is also capable of hydrolyzing 2-AG [8]. Inactivation of one or more of these enzymes can increase steady-state endocannabinoid levels and is one proposed mechanism to limit or reduce inflammation [9,10,11].

Previous work from our lab demonstrated that lipopolysaccharide (LPS) treatment of mice induced pro-inflammatory mediators such as interleukin (IL)-6 and reduced the metabolism of endocannabinoids in the spleen by Ces2g [9]. Several studies using in vivo and ex vivo approaches have shown that 2-AG can suppress pro-inflammatory cytokine levels such as tumor necrosis factor-α (TNF-α), IL-6, and IL-1β by decreasing their production or release [4,11]. For example, mouse macrophages and rat microglial cells treated with exogenous 2-AG exhibited decreased production or release of these cytokines in response to LPS treatment [10,12]. 2-AG was also shown to have a protective role in several disease processes in humans and rodents, such as experimental autoimmune encephalomyelitis (mouse model of multiple sclerosis), colitis, and autoimmune disease [11,13,14]. Treatment of animal models with small-molecule inhibitors that block endocannabinoid-metabolizing enzymes was shown to reduce disease-associated inflammation [3,15,16,17]. For example, inhibition of MAGL by the inhibitor JZL184 following a muscle contusion injury in rats decreased TNF-α, IL-6, and IL-1β levels three days post-injury [18]. Within the context of neuroinflammation, JZL184 decreased inflammatory responses of microglia in the APdE9 mouse model of Alzheimer’s disease when exposed to LPS, IFN-γ, and Aβ_42_ [15]. Interestingly, another study found that IL-6 suppressed CES1 and CES2 mRNA and enzyme activity in primary human hepatocytes [19], providing evidence to support the hypothesis we proposed in our earlier publication [9] that this may be a negative feedback mechanism to regulate 2-AG catabolism and inflammation resolution.

Huntington’s disease (HD) is a progressive neurodegenerative disease caused by a polyglutamine expansion in the human *HTT* gene, which is located on chromosome 4. This mutation results in the aggregation of mutated huntingtin protein in many neuronal cell types, including medium spiny neurons of the striatum [20]. Mutant protein aggregation leads to progressive cell loss and development of motor, cognitive, and psychiatric manifestations of HD over time [21]. Chronic inflammation is observed in both the central and peripheral nervous system in HD, and IL-6 levels have been reported to be elevated in the plasma and cerebrospinal fluid of HD individuals and a mouse model of HD [22,23,24,25,26]. It was also reported that FAAH activity was reduced in blood lymphocytes from HD individuals compared to those from non-HD individuals [27]. Another study in the R6/2 mouse model of HD found a decreased level of FAAH activity in the striatum and increased 2-AG levels in whole brain as compared to control mice. However, no differences in peripheral lymphocyte FAAH activity were observed between R6/2 and control mice [28]. These studies suggest that inflammatory diseases might be associated with decreased activities of endocannabinoid-metabolizing enzymes and that HD disease could be a potential model to determine whether IL-6 can suppress endocannabinoid-metabolizing enzyme activity. Therefore, the goals of this study were to (i) examine the role of inflammation, specifically IL-6, on endocannabinoid-metabolizing enzyme activity in peripheral blood mononuclear cells (PBMCs) obtained from non-HD and HD individuals; (ii) identify the primary 2-AG hydrolytic enzymes in human PBMCs; and (iii) determine the cell specificity of MAGL and CES1 expression in human PBMCs. We hypothesized that HD individuals would have elevated plasma IL-6 levels and decreased PBMC endocannabinoid-metabolizing activity as compared to non-HD individuals. We examined IL-6 levels in plasma obtained from HD and non-HD individuals, assessed IL-6 levels and endocannabinoid-metabolizing enzyme activity in PBMCs isolated from HD and non-HD individuals that were stimulated with inflammogens, and characterized the expression of MAGL and CES1 in PBMCs using immunoblot and flow cytometry.

## 2. Results

### 2.1. IL-6 Quantification in Human Plasma and PBMCs

**Figure 1:**
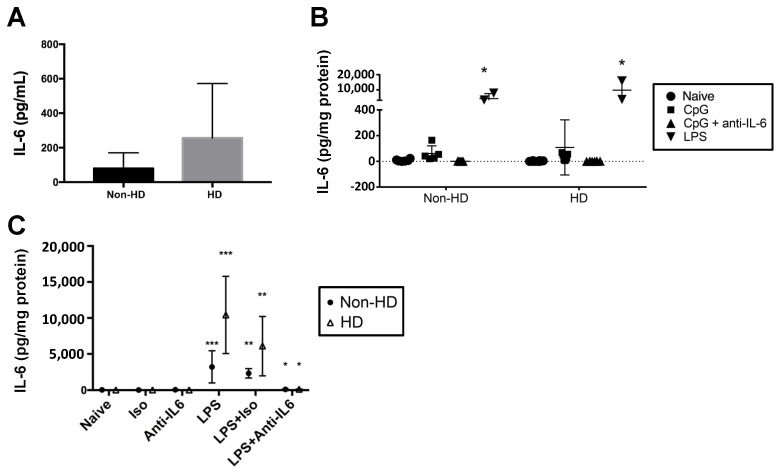
Interleukin-6 (IL-6) levels in non-Huntington’s disease (HD) and HD individuals. (**A**) Plasma was isolated from whole blood of individuals, and plasma IL-6 was measured by ELISA (*n* = 5). (**B**) Peripheral blood mononuclear cells (PBMCs) were stimulated with CpG in the presence and absence of anti-IL-6 neutralizing antibody (*n* = 7 non-HD individuals, *n* = 6 HD individuals). For a few subjects, a comparison between CpG and lipopolysaccharide (LPS) was made to optimize IL-6 stimulation conditions because it was noted that CpG-induced IL-6 levels were relatively low. * *p* < 0.05 as compared to the other treatment groups. (**C**) In follow-up studies, PBMCs from non-HD and HD individuals were stimulated with LPS or were left untreated (naïve) in the presence and absence of IL-6 neutralizing antibody or IgG isotype control; *n* = 6 for all treatments, except for those treatments that utilized LPS alone (*n* = 2). * *p* < 0.05 as compared to LPS + Iso of same treatment group, ** *p* < 0.05 as compared to Iso, *** *p* < 0.05 as compared to Naïve. For (**B**,**C**), IL-6 was detected in supernatants by ELISA and normalized to mg protein in the culture well. Differences between groups and treatments were assessed by Student’s *t*-test (**A**) and two-way analysis of variance (**B**,**C**).

IL-6 levels were determined in plasma obtained from non-HD and HD individuals (Figure 1A); the mean plasma IL-6 level was higher in HD individuals compared to non-HD controls, although it was not statistically significant (*p* = 0.266). IL-6 production by PBMCs from the two groups was also assessed following ex vivo activation with inflammatory mediators (Figure 1B). CpG was found to induce only low levels of IL-6, whereas LPS gave a much more robust response. Therefore, LPS was used instead of CpG to stimulate PBMCs in additional experiments. LPS-induced production of IL-6 from PBMCs did not differ statistically between HD and non-HD individuals (Figure 1C). Importantly, the IL-6 antibody effectively neutralized LPS-evoked IL-6 from the PBMCs (Figure 1C), allowing us to examine the effect of IL-6 on 2-AG hydrolytic activity in subsequent studies.

### 2.2. 2-AG Hydrolytic Activity in Human PBMCs

**Figure 2:**
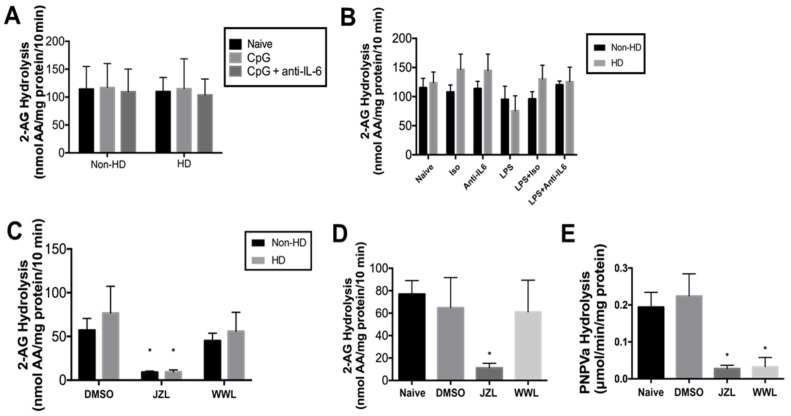
2-arachidonoylglycerol (2-AG) hydrolytic activity and carboxylesterase (CES) activity in human PBMCs. Cell lysates were pre-incubated for 5 min at 37 °C, then supplemented with 2-AG (final concentration, 50 µM). Incubation proceeded for 10 min before quenching the reaction, and arachidonic acid (AA) levels were quantified by LC/MS-MS using AA-d8 as internal standard. PBMCs treated with CpG (1 µM) or CpG with an IL-6 neutralizing antibody were utilized (**A**; *n* = 6) or PBMCs treated with LPS, an IL-6 neutralizing antibody, or an isotype control were utilized (**B**; *n* = 5 for all groups except the group treated with LPS alone, *n* = 2). (**C**) Cell lysates from non-HD and HD individuals (*n* = 5) were pretreated with inhibitors of CES1 (WWL113) and monoacylglycerol lipase (MAGL) (JZL184) (final concentration, 1 µM) or 0.1% DMSO for 30 min at 37 °C prior to adding 2-AG. (**D**) Cell lysates from commercially obtained healthy PBMCs (*n* = 5 individuals) confirmed the results observed with non-HD and HD individuals. (**E**) CES activity of commercially obtained PBMCs (*n* = 5 individuals) was determined by measuring the hydrolysis of the pan-CES substrate pNPVa (final concentration, 500 µM). JZL184 or WWL113 (final concentration, 1 µM) was utilized in some experiments to inhibit CES activity. Three technical replicates were run for each individual. * *p* < 0.05 as compared to DMSO of same treatment group. Differences between groups and treatments were assessed by one-way analysis of variance (**D**,**E**) and two-way analysis of variance (**A**–**C**).

2-AG hydrolysis activity in PBMC lysates was determined by quantifying the amount of AA liberated from exogenously added 2-AG by LC/MS-MS (Figure 2A,B). No differences were noted between HD and non-HD individuals, regardless of whether PBMCs had been stimulated with CpG (Figure 2A) or LPS (Figure 2B). In addition, initial experimentation indicated that the AEA (i.e., FAAH) activity of PBMC lysates (~2 nmol AA produced/mg protein/10 min) was markedly lower than the 2-AG hydrolytic activity (>60 nmol AA produced/mg protein/10 min), so it was not further evaluated in this study.

To characterize the enzymes responsible for the 2-AG hydrolytic activity in PBMCs from non-HD and HD individuals, lysates from untreated (i.e., naïve) cells were pre-incubated with either JZL184—an inhibitor of both MAGL and CES1 [29]—or WWL113—a selective inhibitor of CES1 [30] —prior to adding exogenous 2-AG substrate (Figure 2C). There was no significant change in 2-AG hydrolysis activity in cell lysates pretreated with WWL113 compared to vehicle control, whereas pretreatment with JZL184 significantly reduced 2-AG hydrolysis activity by 81.1 ± 3.9%. No differences in response to the inhibitors were noted between non-HD and HD individuals. The marked inhibition by JZL184 of 2-AG hydrolytic activity without an effect by WWL113 strongly indicates that MAGL is the primary 2-AG hydrolytic enzyme in human PBMCs. Because many of the assessed endpoints were not different in non-HD and HD individuals, we conducted subsequent analyses with commercially obtained human PBMCs. For instance, results using inhibitors of 2-AG hydrolytic activity were similar, with JZL184 significantly reducing 2-AG hydrolysis activity by >6-fold and WWL113 having no effect (Figure 2D). We also verified that WWL113 and JZL184 were functionally active using a colorimetric pan-CES substrate, p-nitrophenyl valerate (pNPVa; Figure 2E). JZL184 and WWL113 were both equally effective at inhibiting CES activity in the PBMC lysates. That WWL113 was unable to inhibit 2-AG hydrolysis whereas JZL184 provides evidence that MAGL is the primary 2-AG hydrolytic enzyme in PMBCs.

### 2.3. CES1 and MAGL Protein Expression in Human PBMCs

**Figure 3:**
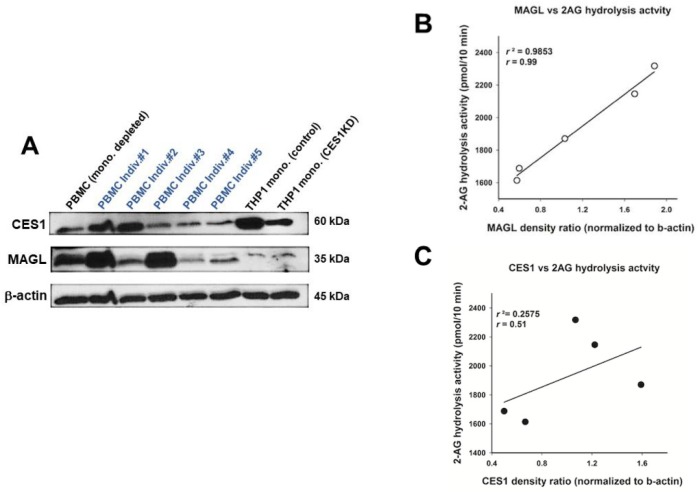
Western blots of CES1 and MAGL in human PBMCs and correlation analysis. (**A**) Commercially obtained PBMC lysates (25 μg of protein per sample; *n* = 5 individuals) and a monocyte-depleted PBMC lysate (25 μg of protein per sample; *n* = 1 individual) were separated by SDS-PAGE and probed with antibodies against CES1, MAGL, and β-actin. β-Actin was used as a loading control. THP-1 monocytic cell lines with high and low levels of CES1 expression (control and CES1 knockdown (KD), respectively; [5]) were used as positive and negative controls. (**B**) The band density for MAGL in each individual (*n* = 5) in the Western blot was correlated with its corresponding 2-AG hydrolytic activity. (**C**) The band density for CES1 in each individual (*n* = 5) in the Western blot was correlated with its corresponding 2-AG hydrolytic activity (25 µg protein per sample for Western and 2-AG hydrolytic activity). Spearman regression correlations were determined for both enzymes. *p* = 0.001 for MAGL; *p* > 0.05 for CES1.

Whereas CES1 is the primary CES isoform expressed in human PBMCs [31] and monocytes in particular [32,33], MAGL activity in human PBMCs has not to our knowledge been clearly documented, although it is expressed in human T cells [34]. To evaluate the expression levels of CES1 and MAGL in human PBMCs, commercially obtained PBMC whole lysates were separated by SDS-PAGE and probed with antibodies that recognize these serine hydrolases (Figure 3A). CES1 and MAGL were expressed in all PBMC samples, and considerable inter-subject variability was noted in their expression levels. CES1 and MAGL were also expressed in monocyte-depleted PBMCs as well as whole PBMCs (Figure 3A), suggesting that lymphocytes could also express these enzymes. Human THP-1 monocytic cell lines (control and CES1 knockdown) were used as controls for CES1 detection [32]. Equal loading of proteins was confirmed by the consistent band density of the β-actin protein between samples.

Next, we performed a correlation analysis between CES1 and MAGL protein amounts in individual PBMCs and their associated 2-AG hydrolytic activities. On the basis of Western blots and enzymatic assays obtained for the PBMCs, a strong positive correlation was observed between MAGL protein abundance and 2-AG hydrolytic activity (r^2^ = 0.9853; Figure 3B), whereas the correlation was weaker between CES1 protein abundance and 2-AG hydrolytic activity (r^2^ = 0.2575; Figure 3C). This correlation analysis provides further evidence that MAGL is the major 2-AG hydrolytic enzyme expressed in human PBMCs.

### 2.4. Monocytes and Lymphocytes Express MAGL and CES1

**Figure 4:**
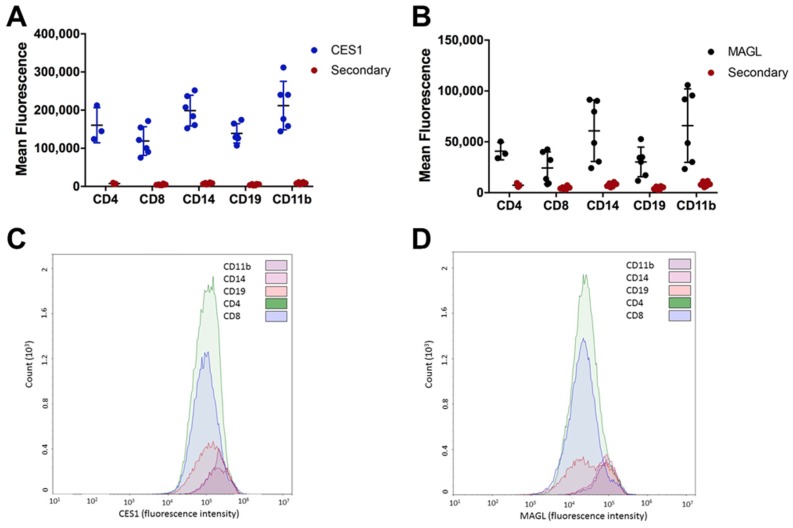
CES1 and MAGL are more highly expressed in monocytes than in lymphocytes. Commercially obtained PBMCs were stained extracellularly for various cell populations (monocytes, CD14 or CD11b), T cells (CD4 or CD8), and B cells (CD19)) and intracellularly for CES1 (**A**,**C**) or MAGL (**B**,**D**). Significantly more fluorescence was detected between the primary antibodies (CES1 or MAGL) than for the secondary antibody alone (**A**,**B**). *p* < 0.5 by two-way analysis of variance for all cell populations except MAGL intracellular staining of CD4^+^ T cells (*p* = 0.1158), CD8^+^ T cells (*p* = 0.2618), and CD19^+^ B cells (*p* = 0.0723). Representative histograms from one donor are provided in (**C**,**D**).

Although we determined that MAGL is the primary 2-AG hydrolytic enzyme expressed in human PBMCs, we also showed that CES1 is present and active in these cells. Thus, we developed a flow-cytometry-based assay to identify the specific cell types in PBMCs that express MAGL and CES1. We assessed the expression of MAGL and CES1 in monocytes (CD14 or CD11b), T cells (CD4 or CD8), and B cells (CD19). MAGL and CES1 expression was relatively higher in monocytes than in lymphocytes (Figure 4A–D). Incubation of cells with the secondary antibody only resulted in a minimal fluorescence signal, demonstrating that the fluorescence from the secondary antibody following CES1 or MAGL staining was specific. Although the absolute fluorescence associated with CES1 expression was higher than that associated with MAGL expression in each cell type, this difference in fluorescence signal does not reflect differences in CES1 and MAGL protein levels because the binding affinity of the secondary antibody for primary antibodies that recognize CES1 and MAGL is most likely different. Thus, we cannot determine whether MAGL or CES1 is the more abundant enzyme in a specific cell type by this analysis; however, it does enable the relative expression of each enzyme across individual cell types to be determined. The major cell types (monocytes and lymphocytes) that comprise PBMCs appear to express both MAGL and CES1, with monocytes expressing more of each enzyme than B and T cells.

## 3. Discussion

Inflammation is a complex process and the roles of endocannabinoids and their biosynthetic and catabolic enzymes in this process are not fully understood. For instance, it is well established that endocannabinoids and inhibitors of endocannabinoid catabolism exert anti-inflammatory effects in some disease processes such as autoimmune disorders, LPS-induced inflammation, and colitis [1,3,10,11,12,13,14,17]. On the other hand, endocannabinoids might be more pro-inflammatory in some disease processes such as atherosclerosis [35]. The role that the endocannabinoid system plays in inflammation resolution is even less understood. Previous work in our laboratory showed that during inflammation which was induced in mice by LPS, there was simultaneous induction of IL-6 levels and inhibition of the activity of the endocannabinoid hydrolytic enzyme Ces2g [9]. These findings suggested the possibility of increased endocannabinoid levels as a negative feedback mechanism to limit inflammation. In this work, we further investigated this possibility using human PBMCs obtained from non-HD and HD individuals by testing the hypothesis that high IL-6 levels observed in individuals with HD could inhibit MAGL and CES activity, thereby increasing or stabilizing 2-AG levels as a potential mechanism to limit inflammation.

It was reported previously that IL-6 levels are increased in the plasma of individuals with HD, and this cytokine has been proposed as a potential biomarker for the onset and progression of the disease [22,25]. In our study, an increased (but nonsignificant) trend in plasma IL-6 levels in HD individuals as compared to non-HD individuals was noted. There are several possible explanations for why our results were not statistically significant. First, only five subjects in each group were evaluated for plasma IL-6 levels. Second, it has been reported in the literature that there is a possibility of alterations in cytokine levels during processing and storage of blood [36,37,38]. Additionally, the sample collection procedure, patient behavior before a blood draw, patient health, and physical activity can all influence cytokine levels [37].

We initially utilized CpG, a form of bacterial DNA that binds to TLR9, to stimulate the human PBMCs to produce IL-6. However, LPS was found to be much more effective than CpG at stimulating IL-6 production. Although there was no significant difference between the IL-6 levels in PBMC supernatants derived from HD and non-HD individuals, there was an increased trend of IL-6 in HD individuals versus non-HD individuals. This trend is consistent with the literature in which IFN-γ-stimulated monocytes from HD individuals and an HD mouse model displayed a more robust immune response than did those from controls [22]. However, we must acknowledge that our studies were limited to assessing IL-6 after one time point (3.5 h), so future studies evaluating additional time points and cytokines would be needed to further explore this.

Regardless of individual type (non-HD or HD), there was no significant difference in 2-AG hydrolytic activity between non-stimulated and stimulated PBMCs. The lack of difference in 2-AG hydrolytic activities in PBMCs from HD and non-HD individuals was in contrast to previous reports that reported that the activity of FAAH, which hydrolyzes AEA, was markedly reduced in the lymphocytes of individuals with HD [27]. Together these results suggest that the catabolic enzymes for 2-AG and AEA in HD individuals could be differentially regulated.

Although we did not identify a role for IL-6 in decreasing endocannabinoid metabolism in this study, increasing 2-AG levels by inactivating enzymes that catabolize it has been shown to induce a protective effect in several disease processes [3,10,13,14,15,16,17,18,39], so it is important to further characterize the 2-AG hydrolytic enzymes in humans. In the present study, we used specific inhibitors of MAGL and CES1 to determine that MAGL is the primary 2-AG hydrolytic enzyme in human PBMCs. Further support for this finding was obtained by correlation analysis of MAGL and CES expression levels with the 2-AG hydrolytic activity in each PBMC sample. Thus, MAGL, and not CES, would be the best therapeutic target in human PBMCs to increase 2-AG levels in circulating immune cells.

Unlike our previous study which showed that Ces2g activity was attenuated in mouse spleen after in vivo LPS exposure [9], the carboxylesterase this study focused on in human PBMCs was CES1, which is orthologous to Ces1d in mice [40]. In contrast to Ces1d, which is poorly expressed in mouse macrophages [41], it is known that CES1 is present in abundance in human macrophages and monocytes [5,6,33]. THP-1 cells, a well-established model of human macrophages, express CES1 but not CES2, and CES1 has been shown to efficiently hydrolyze 2-AG in intact cells [5,6]. However, monocytes only constitute ~5–10% of the PBMC population [42,43], thus possibly limiting their role in 2-AG catabolism by CES1. On the other hand, MAGL has been reported to be expressed in human T cells, which constitute ~60–70% of the PBMC population [34]. In the present study, we confirmed the expression of both CES1 and MAGL in monocytes and lymphocytes by flow cytometry. In agreement with the literature [33], CES1 was highly abundant in monocytes, whereas its levels in lymphocytes were lower. We also determined that MAGL is expressed at higher levels in monocytes as compared to lymphocytes.

The fact that MAGL and CES1 are both expressed in PBMCs but MAGL is the main metabolizing enzyme of 2AG was a somewhat surprising result, especially because the catalytic efficiencies (*k*_cat_/*K*_m_) of recombinant human CES1 and human MAGL toward 2-AG are comparable to each other [6]. The most likely reason is that the 2-AG hydrolytic activities of enzymes within cell lysates are different from those of purified enzymes. It is possible that accessory proteins and/or lipid membranes that MAGL and CES1 associate with in cell lysates influence their ability to hydrolyze the 2-AG substrate (i.e., affect their catalytic efficiency). The topology of the enzymes within the cell (and the resulting cell lysates) might also play a role. For example, CES1 is found within the ER lumen tethered to integral membrane proteins called KDEL receptors via its C-terminal ER retention signal peptide [44], whereas MAGL is loosely associated with plasma and endoplasmic reticulum membranes on the leaflet facing the cytoplasm [45]. The way these enzymes are oriented may influence their interaction with exogenously added 2-AG substrate.

Although this study is limited in the number of individuals that were evaluated, several important observations were made. First, MAGL and CES1 were both detected in human PBMCs from both non-HD and HD individuals. Second, the levels of endocannabinoid enzymes expressed in human PBMCs are variable. Third, MAGL and CES1 are more highly expressed in monocytes as compared to lymphocytes. Finally, MAGL is the predominant enzyme for 2-AG hydrolysis in human PBMCs and a potential target of inhibitors to increase levels of 2-AG in circulating immune cells.

## 4. Materials and Methods

### 4.1. Chemicals and Reagents

Histopaque, p-nitrophenyl valerate (pNPVa), and LPS (*E. coli* 055:B5) were purchased from Sigma (St. Louis, MO, USA). AIM V^®^ Serum Free Medium was purchased from Thermo-Fisher (Waltham, MA, USA). RIPA lysis buffer and protease inhibitors (phenylmethylsulfonyl fluoride, PMSF; 4-(2-aminoethyl)benzenesulfonyl fluoride, AEBSF; bestatin; pepstatin A; leupeptin hemisulfate; and aprotinin) were from Santa Cruz Biotechnology (Dallas, TX, USA). Antibodies used for Western blots (anti-CES1, anti-MAGL, β-actin, goat anti-rabbit, and goat anti-mouse) were purchased from Abcam (Cambridge, MA, USA). Authentic 2-AG, anandamide, AA, and its deuterated analog AA-*d*8 were purchased from Cayman Chemical Company (Ann Arbor, MI, USA). Small molecules JZL184 and WWL113 were purchased from Sigma. CpG was purchased from InvivoGen (San Diego, CA, USA). Primary CES1 and MAGL antibodies for flow cytometry were purchased from Abcam. Fluorescence secondary antibodies for flow cytometry and antibodies against IL-6 used to neutralize IL-6 or perform ELISA were purchased from Biolegend (San Diego, CA, USA). For some of the experiments, monocyte-depleted (*n* = 1) and whole PBMCs (*n* = 5) were purchased from Astarte Biologics (Bothell, WA, USA).

### 4.2. Blood Collection

Human venous blood was collected in EDTA Vacutainer tubes from 8 non-HD individuals and 8 symptomatic HD individuals at the University of Rochester (Rochester, NY, USA). Blood was shipped at room temperature (RT) overnight to Mississippi State University for analysis. All study-specific procedures were approved by the University of Rochester Research Subject Review Board and the Mississippi State University Institutional Review Board.

### 4.3. PBMC Isolation and Culture

PBMCs were isolated from whole blood utilizing Histopaque density gradient centrifugation. PBMCs (average yield was 90 million cells per subject) were resuspended in AIM V^®^ Serum Free Medium and divided equally into 10–12 wells at a volume of 4 mL each. Cells were either left untreated (naïve) or treated for 3.5 h at 37 °C with an isotype control (IgG; 0.5 mg/mL), anti-IL-6 to neutralize levels of extracellular IL-6 (0.5 mg/mL), LPS (1 µg/mL), LPS plus anti-IL-6, LPS plus IgG, CpG (1 µM), or CpG plus anti-IL-6. After the culture period was over, supernatants were collected for ELISA; cells were separated from the medium by centrifugation and washed with 1 mL of PBS. The cells and supernatants were stored at −80 °C.

### 4.4. Determination of IL-6 Levels by ELISA in Supernatants and Plasma

Before isolating PBMCs, 500 µL of whole blood was transferred to a BD microtainer separator tube and centrifuged at 3000× *g* for 15 min. The resulting plasma was stored at −80 °C. An ELISA plate containing 100 µL of a 1:500 dilution of anti-human IL-6 antibody (Biolegend) in ELISA wells was incubated overnight at 4 °C. The plate was then rinsed three times each in 3% BSA in PBS and distilled water, and the plate was blocked for 1 h at RT in 3% BSA in PBS. The plate was rinsed again and 50 µL or 100 µL of IL-6 standards, 50 µL of plasma, or 100 µL of PBMC culture supernatant was added to each well for a 1 h incubation. After rinsing, 100 µL of a 1:500 biotinylated anti-human IL6 antibody (Biolegend) was added for a 1 h incubation. The plate was rinsed again, and 100 µL of 1:1000 horse radish peroxidase (HRP)-Avidin (Biolegend) was added to each well and incubated for 30 min. In the next step, 100 µL of a 1:1 tetramethylbenzidine (TMB) solution (Thermo-Fisher) was added to each well after a rinse. The reaction was allowed to incubate for 15 min at RT and was stopped with 100 µL of 2 N H_2_SO_4_ before reading on a plate reader at an absorbance of 450 nm.

### 4.5. Cell Lysate Preparation and Protein Determination

PBMCs were resuspended in 100 µL of 50 mM Tris-HCl (pH 7.4) and lysed by sonication (3 × 10 sec intervals) on ice. Lysed samples were centrifuged at 1000× *g* at 4 °C for 10 min to remove cellular debris, and the supernatants were stored at −80 °C until utilized further. The lysates were diluted 1:5 *v*/*v* (25 µL total volume) in deionized water and incubated with bicinchoninic acid (BCA) working reagent (200 µL) for 30 min at 37 °C. Absorbance was measured at 560 nm on a plate reader and compared to a bovine serum albumin standard to determine the protein concentrations of the samples.

### 4.6. Preparation of Commercially Obtained PBMCs

After receiving the commercial PBMCs, they were stored at −150 °C until processing. Each sample was thawed quickly at 37 °C and the cells washed with 9 mL of 1× PBS. The samples were centrifuged at 500× *g* for 5 min before removing the supernatant. The washed cells were resuspended in 2 mL of PBS and divided equally into two microcentrifuge tubes. The samples were then centrifuged at 16,100× *g* for 5 min and washed again in 1 mL of PBS. One aliquot of the cells used for Western blots was lysed in 250 µL of RIPA lysis buffer containing protease inhibitors. After a 30 min incubation on ice, the lysed cells were further sonicated on ice (3 × 10 sec intervals) and then divided into 80 µL aliquots for storage at −80 °C. The second aliquot of cells used for enzyme activity assays was resuspended in 250 µL of 50 mM Tris-HCl (pH 7.4) and sonicated on ice (3 × 10 sec intervals) and then divided into 80 µL aliquots for storage at −80 °C.

### 4.7. 2-AG Hydrolysis Activity of PBMC Lysates

A quantity of 10 µL of PBMC lysate (prepared in Tris buffer) was diluted to a final volume of 50 µL with 50 mM Tris-HCl (pH 7.4) and pre-incubated for 5 min at 37 °C. Authentic 2-AG was then added (final concentration of 50 µM), and the sample was incubated for 10 min at 37 °C before quenching the reaction with 100 µL of cold acetonitrile (containing 2.5 µM of AA-*d*8). The samples were stored on ice for at least 10 min before centrifugation at 16,100× *g* for 10 min at 4 °C. A quantity of 100 µL of supernatant was transferred to HPLC vials with glass reducing inserts and the vials stored at −20 °C until analysis. Non-lysate controls containing 50 µL of 50 mM Tris-HCl (pH 7.4) were also prepared in an identical manner. To ensure that levels of endogenous AA in cell lysates did not affect results, samples were prepared without the addition of authentic 2-AG. In some experiments, cell lysates were pre-incubated for 15 min at 37 °C with 0.1% DMSO (vehicle control), JZL184 (1 µM), or WWL113 (1 µM) prior to adding 2-AG. Analysis of AA levels was performed by LC/MS-MS as described; peak areas of AA were normalized on the AA-*d*8 internal standard peak area and protein amount [46]. 2-AG hydrolysis rates are reported as nmol or pmol AA formed/mg protein/10 min. At least two technical replicates for each PBMC sample were performed.

### 4.8. Determination of Carboxylesterase Activity by pNPVa Hydrolysis Assay

PBMC lysates were diluted in 50 mM Tris-HCl (pH 7.4) and pre-incubated for 5 min at 37 °C. pNPVa in ethanol stock was added to the mixture to give a final concentration of 500 μM. The production of para-nitrophenol was evaluated continuously for 5 min at 405 nm, and the calculated enzyme activity was corrected by the protein concentration of the sample. In some experiments, lysates were pre-incubated for 5 min at 37 °C with 0.1% DMSO (vehicle control), JZL184 (1 µM), or WWL113 (1 µM) prior to adding pNPVa. Experiments were performed in technical triplicates.

### 4.9. Western Blot Analysis

PBMC lysates (25 μg per sample) were resolved on 10% SDS-PAGE gels and transferred to a PDVF membrane. CES1 and MAGL proteins were detected by incubation with rabbit monoclonal anti-human CES1 (1:25,000 *v*/*v*) or mouse monoclonal anti-human MAGL (1:10,000 *v*/*v*), followed by incubation with goat anti-rabbit or goat anti-mouse secondary (1:8000 *v*/*v*). β-Actin was detected with an anti-β-actin antibody to ensure equal loading of protein. Blots were visualized on film using enhanced chemiluminescence using ThermoSupersignal West Pico ECL reagent. The resulting films were scanned, and densitometry analysis was performed using ImageJ v1.49a (NIH, Bethesda, MD, USA).

### 4.10. Flow Cytometry

PBMCs (1 million per well) were stained with Near-IR fixed viability dye (Biolegend) before the Fc receptors were blocked with TruX stain. Cells were then stained with extracellular antibodies for CD4 (FITC), CD8 (PECy7), CD11b (PECy5), CD14 (APC), CD19 (BV650), and CD3 (PacBlue). Following extracellular staining, cells were fixed, permeabilized, and incubated with a primary antibody for CES1 or MAGL for 30 min at RT. Cells were then incubated with a secondary antibody conjugated to PE for 30 min and analyzed on an ACEA Novocyte Flow Cytometer. Antibody capture beads (eBioscience) were used to set compensation, and fluorescence minus one controls were used to set gates. A secondary-only control was used to verify specific staining for CES1 and MAGL. However, because the same secondary antibody was used to detect CES1 and MAGL, each sample had to be analyzed separately and the results are presented as mean fluorescence intensity as compared to the secondary-only control for each protein.

### 4.11. Statistical Analysis

The mean and standard deviation (or standard error) were calculated for each experimental group. Student’s *t*-test and one-way or two-way analysis of variance were used to assess differences between groups or treatments with GraphPad Prism Software (Version 7, San Diego, CA, USA) when appropriate. Spearman regression correlations between 2-AG hydrolytic data and Western blot results were determined using SigmaPlot Software (Version 11.0, San Jose, CA, USA). A *p*-value of <0.05 was considered significant.
